# Performance of Sustainable Insulated Wall Panels with Geopolymer Concrete

**DOI:** 10.3390/ma15248801

**Published:** 2022-12-09

**Authors:** Balamurali Kanagaraj, Tattukolla Kiran, Jayakumar Gunasekaran, Anand Nammalvar, Prince Arulraj, Beulah Gnana Ananthi Gurupatham, Krishanu Roy

**Affiliations:** 1 Department of Civil Engineering, Karunya Institute of Technology and Sciences, Coimbatore 641114, India; 2 Department of Civil Engineering, Sri Krishna College of Technology, Coimbatore 641042, India; 3 Division of Structural Engineering, College of Engineering Guindy Campus, Anna University, Chennai 600025, India; 4 School of Engineering, The University of Waikato, Hamilton 3216, New Zealand

**Keywords:** expanded polystyrene, geopolymer concrete, sustainability, masonry, axial strength

## Abstract

The increase in the population creates an increased demand for construction activities with eco-friendly, sustainable, and high-performance materials. Insulated concrete form (ICF) is an emerging technology that satisfies the sustainability demands of the construction sector. ICF is a composite material (a combination of expanded polystyrene (EPS) and geopolymer concrete (GPC)) that enhances the performance of concrete (such as thermal insulation and mechanical properties). To investigate the axial strength performance, five different types of prototypes were created and tested. Type I (without reinforcement): (a) hollow EPS without concrete, (b) alternative cells of EPS filled with concrete, (c) and all the cells of EPS filled with concrete; and Type II (with reinforcement): (d) alternative cells of EPS filled with concrete; (e) and all the cells of EPS filled with concrete. Amongst all the five prototypes, two grades of GPC were employed. M15 and M20 grades are used to examine the effectiveness in terms of cost. For comparing the test results, a reference masonry unit was constructed with conventional clay bricks. The main aim of the investigation is to examine the physical and mechanical performance of sandwich-type ICFs. The presence of polystyrene in ICF changes the failure pattern from brittle to ductile. The result from the study reveals that the Type II prototype, i.e., the specimen with all the cells of EPS filled with concrete and reinforcement, possesses a maximum load-carrying capacity greater than the reference masonry unit. Therefore, the proposed ICF is recommended to replace the conventional load-bearing system and non-load-bearing walls.

## 1. Introduction

Expanded polystyrene (EPS) is considered one of the common materials used instead of aggregates to reduce weight and for improving thermal comfort [1]. EPS is a kind of stable foam with low density, consisting of discrete air voids in a polymer matrix [2]. As a lightweight artificial aggregate, EPS is commercially available and can be incorporated in mortar or concrete to produce lightweight insulating concrete [3]. From the engineering point of view, the advantage of using EPS among different types of LWAs is the low water absorption due to its lower porosity. In addition, the low thermal conductivity of EPS (0.03–0.04 W/mK) is one of the main reasons for its use in the construction industry, particularly for insulting purposes [4]. Previous research has promoted the applications of EPS-based concrete in construction and building products. Currently, EPS lightweight concrete is used in various structural and non-structural elements such as precast concrete panels, cladding panels, composite flooring systems, subbase materials in pavements, floating marine structures, and insulating building elements [5]. However, replacing the normal-weight aggregate with EPS lightweight aggregate increases the drying shrinkage strain [6,7].

Due to the over-exploitation of virgin construction and building materials, there is a need to develop a suitable alternative eco-friendly building material to satisfy the demand for infrastructural activities. EPS is found to be one of the alternatives fabricated of recycled materials; since EPS is a non-degradable material, it can be recycled after its first life, second life, and so on [8]. EPS is recycled either in the form of beads or modified with heat treatment [9]. Recycling and using such materials in the construction sector protects the environment by preventing the emission of harmful gases when incinerated.

Quality construction practices are gaining demand with the increase in the population [10]. ICF is found to be a promising construction practice. ICF walls are fabricated by placing concrete in the individual cells of EPS foam. Construction of ICF structures has many advantages over conventional structures based on cost, ease of construction practices, thermal insulation, etc. Some other benefits are heatproof, soundproof, faster construction, lower maintenance, and resistance against insects, wind, and disasters [11]. In the United States (US), nearly 3% of houses are constructed using ICF systems [12]. ICF is one of the sustainable, eco-friendly alternatives to conventional carbon-emitting cement-based construction practices. ICF’s superior performance is mainly due to the EPS [13]. ICFs are used as construction materials in the construction of schools, colleges, hospital buildings, etc. ICF claims several advantages over conventional building materials [14]. ICF has been a boon to the construction sectors in India since 2013; ICF-based structures have been accepted in the US, Germany, Japan, Canada, and Mexico. Moreover, the construction cost of ICF structures is 5–10% less than conventional practices. ICF construction practices require more awareness to accept this new system of the construction sectors to meet the current requirements of the infrastructure industry.

EPS employed for producing ICF should have a density of 20 kg/m^3^ to 40 kg/m^3^ and a thickness of 50 mm to 100 mm. ICF panels are generally employed to construct a wall system to portion the functional area with a structural wall system. The wall systems are mainly subjected to axial compression due to the load transfer from the floor system. The main intention of the axial load test is to determine the ultimate load-carrying capacity, failure pattern, and stress–strain relationship, which helps to design the ICF panels. Past studies have reported that axial compression tests are performed on different wall panels, such as foamed wall panels, composite panels, reinforced panels, precast sandwich panels, precast foamed panels, panels with openings, and panels with shear connectors. Similarly, a four-point bending test was performed on bridge deck panels, Ferro-cement panels, lightweight panels, fiber-reinforced sandwich panels, and hollow concrete wall panels.

However, OPC production requires argillaceous and calcareous materials and is energy-intensive. The main reasons for the emission of greenhouse gas during the production of OPC are calcination and fossil-fuel combustion [15,16,17]. The production of conventional Portland cement increases yearly by about 8% to 10%. These cement types account for 8% of global carbon dioxide emissions (CO_2_-e) [18]. To reduce global CO_2_-e, an alternative binder must be used. Supplementary cementitious materials (SCMs) such as sugarcane bagasse ash, Metakaolin (MK), red mud, and Ground Granulated Blast Furnace Slag (GGBS) are industrial by-products. SCMs have cementitious properties and are employed as a cement alternative [19,20]. A durable structure with less greenhouse gas emission and with less energy can be obtained by the addition of fly ash to the concrete [21,22,23,24]. Investigations are carried out on SCMs for producing sustainable, eco-friendly concrete. The outcomes of using SCMs as partial cement replacements are gaining economic impact in reducing CO_2_-e during its production.

In 1978, a novel binding medium was named geopolymer, which employs 0% cement in its production. These geopolymers are a suitable alternative that completely replaces conventional Portland cement concrete. The early age strength of geopolymer is higher than that of traditional cement concrete and possesses higher fire resistance. Moreover, these SCMs can replace cement by 20% to 30% by weight, beyond which the strength reduction is more significant. The dissolution makes the production of geopolymer concrete (GPC) of source material possible with the alkaline solution (combination of NaOH and Na_2_SiO_3_) [25]. The source material can be either from geological origin or industrial by-products such as GGBS, MK, Palm Oil Fuel Shell Ash (POFA), Rice Husk Ash (RHA), Fly Ash (FA), and so on [26]. Previous research has shown that GPC concrete has superior performance compared with OPC concrete in terms of mechanical and durability characteristics [25,27].

A combination of GPC and ICF can form a sustainable construction practice. The proposed system’s significant advantages are lightweight, ease of placement, faster construction, optimized cost, strength, and eco-friendly. Reduction in cost and CO_2_ emissions are some of the indicators that might be helpful to achieve sustainability goals. The effective utilization of GPC in the ICF system can improve the load-withstanding capacity of the wall panel. With this background, the current study aims to analyze the load-deformation behavior of GPC-ICF systems subjected to axial compressive loading.

Insulated concrete form (ICF) panels are structural wall panels fabricated by pouring concrete in interlocked expanded polystyrene (EPS) that hold the concrete together during the curing process. The EPS form is a permanent part of a wall panel and provides thermal insulation to the building, whereas the reinforced concrete affords a structural system to the building [12]. Applications of ICFs are extended to a wide range of building constructions, including residences, theatres, schools, and hospitals [13]. EPS is a by-product of the petroleum industry and is derived by the styrene hydrocarbon polymer (polystyrene) expansion using pentane gas. An EPS bead consists of 2% raw material and 98% of air, which is chemically composed of two elements: carbon and hydrogen [11]. Generally, EPS sheets have been used in various applications, including impact mitigation packaging, protective helmet, expansion joints, construction filling material, false ceilings, and food packaging material. Diverse structural and geotechnical applications of EPS are also found in the literature, namely structural insulated panels [28], composite structural insulated panels [5], insulated concrete sandwich panels [14], lightweight concrete sandwich panels [29], and thermal insulators [2].

The past study found that the axial compression test was performed on various panels of fibers, concrete, and EPS sheets. Research shows that the wall panel characterization focused on load-deflection behavior and load-carrying capacity of the structural element. Moreover, fewer types of research are available on the ICF wall panels subjected to axial compressive loads. In the present work, the ICF wall panels are constructed by creating a hollow outer shell and filling the hollow core with concrete. The study’s main aim is to examine the ultimate load-carrying capacity of ICF, load-deflection behavior, and failure pattern of ICF wall panels with GPC filling. The findings may be helpful for future work with large-scale models.

## 2. Materials and Methods

### 2.1. Binder

Class F Fly Ash (FA) and GGBS were the source materials obtained from the local market. The chemical composition of the binder material is illustrated in Table 1 [30].

#### 2.1.1. Filler

**Figure 1:**
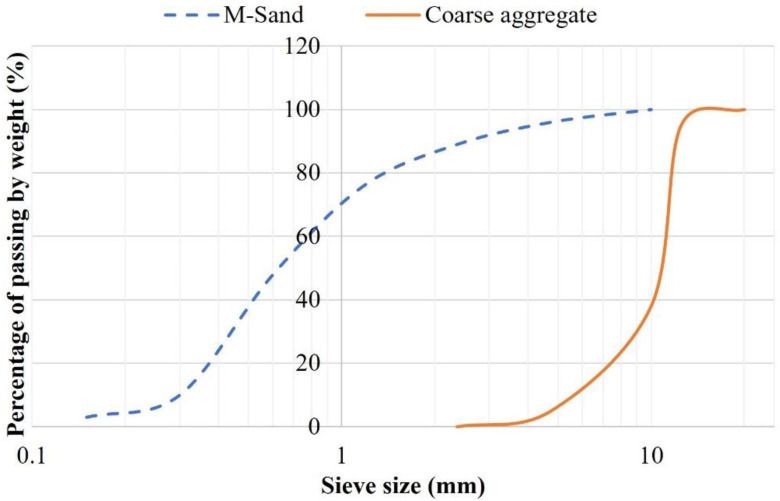
Particle size distribution of M-Sand and coarse aggregates.

Locally available manufactured sand (M-sand) and crushed granite chips were employed as the filler materials (fine and coarse aggregates) for the production of GPC. The specific gravity, water absorption, and fineness modulus of M-sand were found to be 2.75, 1.37%, and 2.59, respectively. The specific gravity and water absorption of coarse aggregate were found to be 2.81 and 0.92%, respectively. The size of the M-sand employed in the present investigation falls between 4.75 mm and below, whereas coarse aggregate is between 12.5 mm and 20 mm, confirming IS 383 [31]. Figure 1 illustrates the semi-log graph of M-sand and coarse aggregate.

#### 2.1.2. Activator Solution (AS)

A synthesis between an alumino-silica source and an alkaline activator solution [13], yields an alumino-silica source and an alkaline activator solution (AS). AS is a combination of NaOH (NH) and Na_2_SiO_3_ (NS) [32,33]. NH and NS are obtained from local dealers in Coimbatore. In general, the NH is available in the form of flakes or pellets, and it must be made into a solution by dissolving the required amount of NH pellets or flakes in water. NS is available in the form of liquid and can be used directly.

#### 2.1.3. Expanded Polystyrene (EPS)

EPS is a combination of air and styrene, and the whole wall shell system is fabricated of 98% air and 2% styrene. Due to the advantage of the EPS shell, the ICF wall panels retain their shape even after failure. Table 2 illustrates the material properties of an ICF wall.

#### 2.1.4. Mix Design

As mentioned above, AS is a combination of NH and NS. The NH concentration employed in the present investigation is four molarity, and the ratio of AS used is 1:1.5 [34]. The pellets or flakes of NH are to be made into a solution before concrete production [35]. The AS was made one day before the casting to initiate the polymerization reaction [25]. The mix design carried out in the study was entirely based on the trial-and-error method. Table 3 illustrates the recipe for the mix involved in the current investigation.

### 2.2. Methods

#### 2.2.1. Specimen Preparation

**Figure 2:**
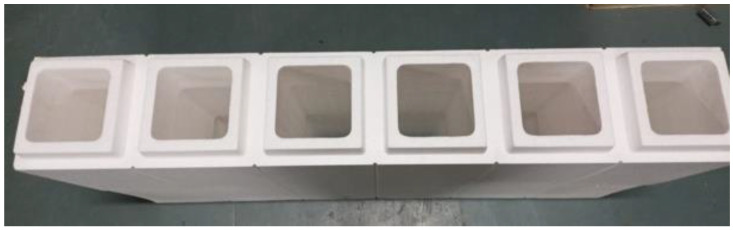
Structure of the EPS shell.

**Figure 3:**
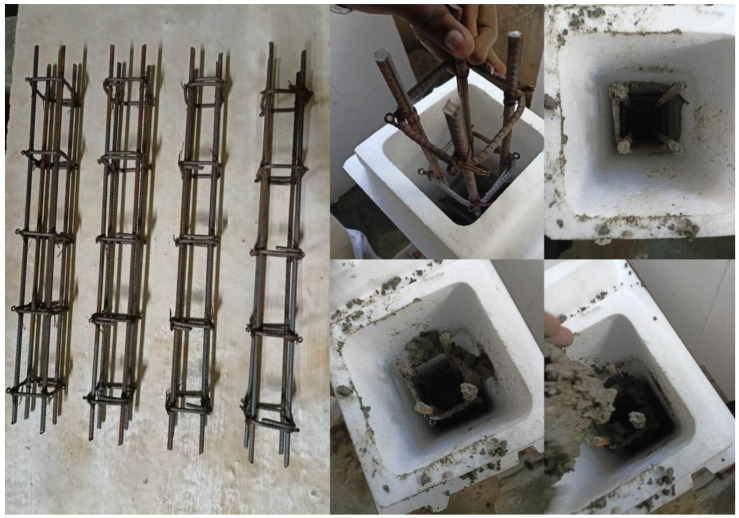
Reinforcing ICF wall.

**Figure 4:**
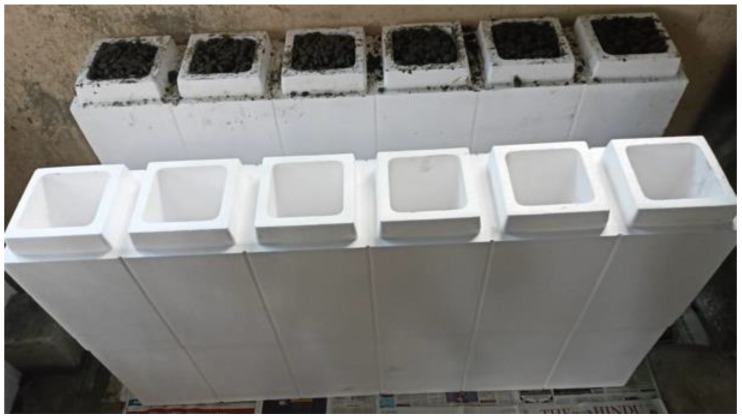
The casting of the ICF.

**Figure 5:**
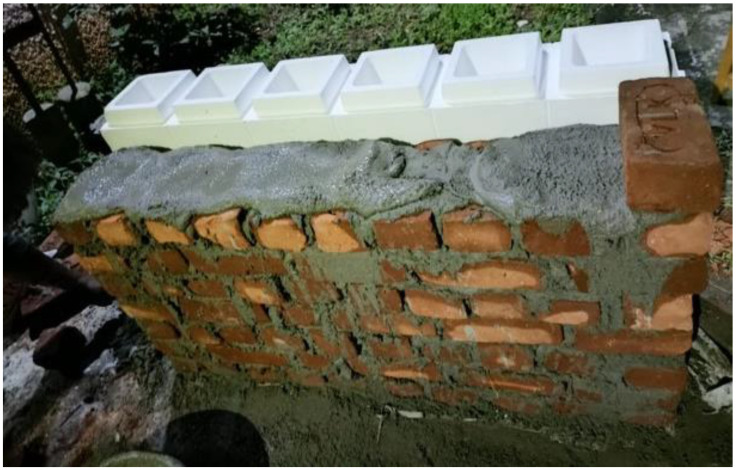
Construction of the conventional brick masonry.

The filler materials, fine and coarse aggregates, were mixed in the rotary-type mixer drum for 1–2 min. Then binder materials FA and GGBS were added to the mixer drum, and the drum was rotated for 2–3 min to achieve uniformity in the mix. After attaining uniformity in the mix, the prepared AS mix was added; then the mixture was rotated for another 2 min to achieve homogeneity [35]. After preparing the mix, fresh properties were tested on the prepared mix to check its workability. After checking the slump test, the freshly prepared mix was poured into the EPS shell’s hollow portion. The mix was set at room temperature for 24 h to initiate the polymerization reaction process. After setting, the ICF (combination of EPS and GPC) was cured at a room temperature of 27 ± 2 °C for 28 days to achieve the target strength. At the end of 28 days of the curing period, the ICF was tested to examine the load-carrying capacity. Table 4 shows the details of specimens prepared for the study. Figure 2 represents the outer shell of the EPS form. Figure 3, Figure 4 and Figure 5 represent the casting of ICF panels and masonry reference units. For better performance with respect to durability of the steel reinforcement stainless steel of various types (Ferritic, Duplex, and Austenitic) can also be used [36].

#### 2.2.2. Workability Test

The freshly prepared mix was tested for its workability using a slump cone. A slump test was performed to assess the fresh property of the GPC. A 300 mm high slump cone was employed in the present investigation to assess the fresh property. Waste lubricant was applied on the inner side of the cone initially to prevent the sticking of concrete with the cone. Then, the mold was filled with concrete in three layers with the compaction of 25 rods for each layer, after filling the cone with the freshly prepared concrete. The excessive layer at the top of the mold was trimmed, and slowly, the mold was lifted. The difference in GPC height and the mold is represented as the slump value [37].

#### 2.2.3. Test Setup for Axial Compression Test on ICF

**Figure 6:**
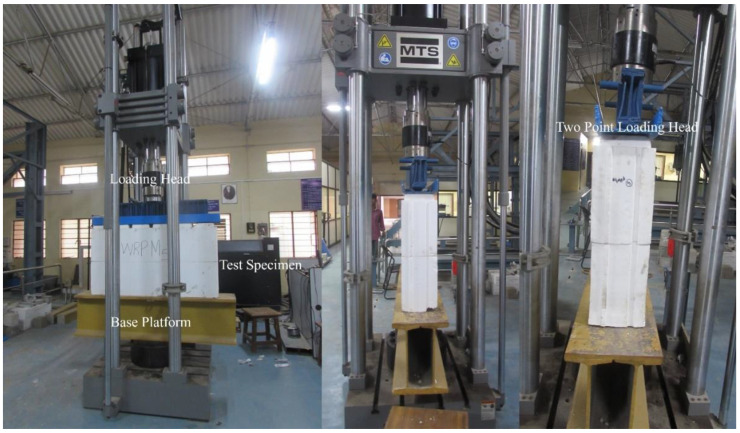
Load testing setup of ICF in MTS (axial compression tests).

The axial compression test on the ICF was conducted with a Servo hydraulic UTM of 100 T capacity (MTS). The MTS system is operated based on displacement control; the displacement rate is controlled during the loading. The ICF’s maximum load-carrying capacity and load-deformation behavior are recorded for every 0.01 mm displacement. The ICF panels are positioned in the vertical direction of the MTS system. A 20 mm thick plate and the spacer bars are provided at the top of the wall panel to distribute the load uniformly throughout the specimen. Above that, a two-point loading platform is provided to transfer the load from the MTS system to the 20 mm thick plate as shown in Figure 6.

## 3. Results and Discussion

### 3.1. Workability

As per ASTM C 143 [37], the fresh property test on freshly prepared concrete is conducted. A slump cone test was conducted immediately after preparing the mix to determine the workability of freshly prepared geopolymer concrete. It is found from the test results that the M15 mix possesses a target slump of 78 mm, and the M20 mix retains an 85 mm slump.

### 3.2. Compressive Strength (CS)

**Figure 7:**
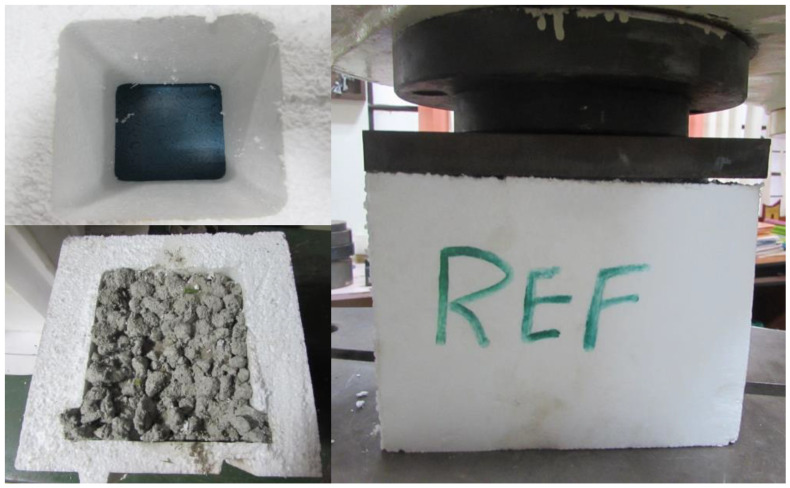
Compressive strength test setup of EPS shell and composite unit (EPS with concrete).

**Figure 8:**
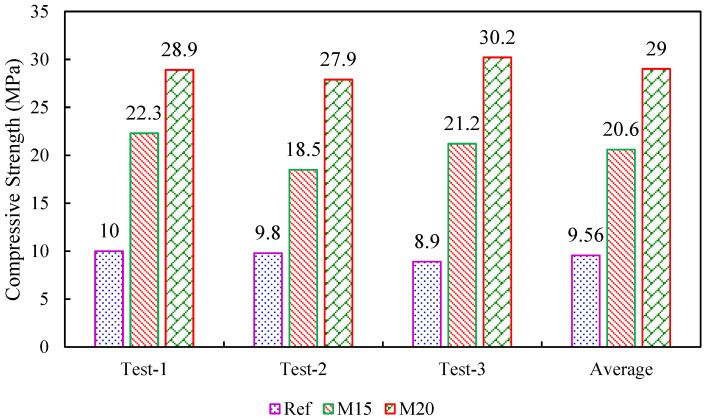
Compressive strength results of EPS with M15 and M20 concrete mixes.

The CS test was performed on a hollow EPS shell and EPS shell (with M15 and M20 concrete) using a computerized UTM of 100 T capacity following IS 516:2004 [38], as shown in Figure 7. The CS test was performed on the 28-day cured GPC specimens. The reference specimen (EPS shell) possesses a maximum average CS of 9.56 MPa. The EPS shell with M15 geopolymer concrete contains an average CS of 20.6 MPa, and the EPS shell with M20 geopolymer concrete possesses an average CS of 29 MPa. Higher strength development of geopolymer concrete was attributed to the presence of GGBS content. From Figure 8 it is clear that an increase in the GGBS content increases the CS of the geopolymer concrete. The curing of geopolymer concrete does not require water, so the curing of geopolymer mix is initiated at room temperature conditions. The strength development of the mix is mainly attributed to the polymerization reaction of the alumina-silica source with the alkaline solutions. The load was applied at the top of the geopolymer concrete blended ICF system. Initially, the geopolymer concrete present in the core of the ICF system reaches its ultimate strength and tends to collapse completely by splitting the concrete into pieces. The concrete was no longer strong enough to withstand the additional load, but the EPS outer periphery contributed to unite the system and prevent wall failure.

### 3.3. Axial Load Deformation of ICF

The axial load test was performed on five types of ICF wall panels. The testing of ICF wall panels is depicted in Figure 6. From the test, it was found that (S1) hollow EPS without concrete failed by crushing, and no sign of buckling was seen in this failure type; and when the load was released, the EPS shell regained its original shape, similar results were found by the researchers [12]. All the other types of panels possess a similar kind of failure; during the test, it was ensured that no load was applied eccentrically on the top of the panel. The original shape of the ICF is not degraded on loading the ICF and under deformation; while releasing the load, it regains its original shape. The elastic nature of the outer shell adds advantages to the concrete present in the core of the wall panel. On the contrary, the failure pattern of the concrete was not visible due to the outer shell of the EPS.

**Figure 9:**
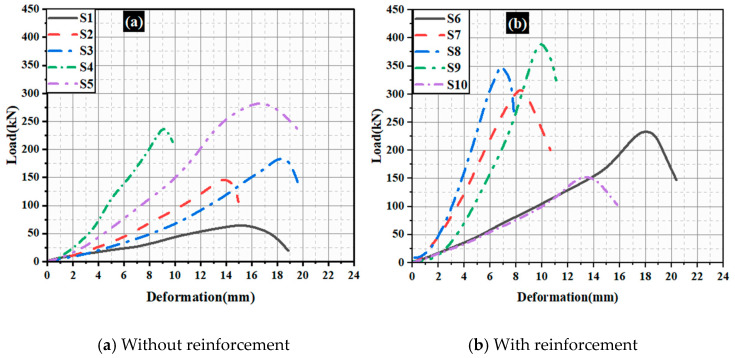
Load–axial-deformation behavior of ICF wall panels and masonry units.

Figure 9 depicts the load-deformation graph of ICF wall panels and masonry units. Specimen S1 (hollow EPS without concrete) exhibits the lowest load of 53 kN with a deformation of 19 mm. Specimen S2 (alternative cells of EPS were filled with concrete without reinforcement, M15) shows a load of 149 kN with a deformation of 15 mm. Specimen S3 (all the cells of EPS were filled with concrete without reinforcement, M15) exhibits a load of 176 kN with a deformation of 20 mm. To understand the loading capacity of the EPS panel under axial compressive loading, the concrete is filled in the voids of the EPS. This is also to optimize the use of concrete in the EPS cells. Lower-grade concrete is preferred to reduce the preparation cost. Specimen S4 (alternative cells of EPS were filled with concrete without reinforcement, M20) demonstrated a load of 230 kN with deformation of 10 mm. Specimen S5 (all the cells of EPS were filled with concrete without reinforcement, M20) showed a load of 260 kN with a deformation of 20 mm. It is observed that the EPS with M20 grade has shown a higher load with less deformation. This is seen in the specimen with the GPC filled in all cells. The reinforcements in the wall system improve the compressive strength and bending capacity. The reinforcements were added to increase the strength and stability of the structural wall system. Four vertical bars of 8 mm diameter and five ties of 6 mm diameter were employed in the present investigation. The ties were provided at a constant interval of 150 mm. The reinforcement was provided in each cell of the wall panel to measure the maximum load-carrying capacity of the reinforced ICF-based wall panels. This also connects the EPS panels as an anchorage to form continuity. The details of load-carrying capacity and axial deformation of EPS panels with GPC and reinforcements are as follows: Specimen S6 (alternative cells of EPS were filled with concrete, M15) exhibits a load of 226 kN with deformation of 20 mm. Specimen S7 (all the cells of EPS were filled with concrete, M15) indicates a load of 300 kN with deformation of 11 mm. Specimen S8 (alternative cells of EPS were filled with concrete, M20) shows a load of 350 kN with a deformation of 8 mm. Specimen S9 (all the cells of EPS were filled with concrete, M20) exhibits a load of 360 kN with a deformation of 11 mm. Specimen S10 (reference masonry unit) shows a load of 150 kN with deformation of 16 mm.

The above test results show that the specimens without reinforcement possess higher deformation than those with reinforcement. Moreover, an increase in the grade of concrete increases the ultimate failure load and decreases the deformation of the ICF wall panels. Specimen S6 possesses a maximum deformation of 20.5 mm; this can be attributed to hollow, empty cells in the ICF wall panels. Moreover, rapid failure was seen in the brick masonry wall. When the ultimate load is reached, the brick masonry walls crumbles; this type of failure is avoided in ICF wall panels. The presence of an EPS shell can be attributed to this. The elastic nature of the EPS shell holds the GPC at the core even after failure. Sudden failure (brittle) was seen in the conventional brick wall. In the present investigation, ICF-based wall panels do not show brittle failure in all cases when they are subjected to loading.

**Figure 10:**
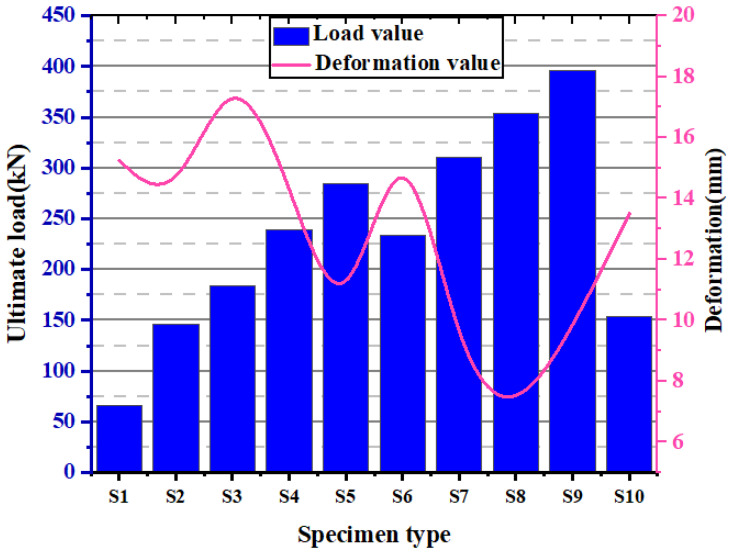
The ultimate load carried by the ICF wall panels, along with their deformation.

The ultimate load of all the ICF wall panels is higher than that of conventional brick masonry. Furthermore, after reaching the ultimate load, the load is removed, and the EPS system holds the crushed concrete inside the core and prevents the failure mode (brittle) of GPC. The load vs. deflection behavior of conventional masonry and ICF-based walls is depicted in Figure 10.

In general, concrete is strong in compression and weak in tension. Once concrete reaches its ultimate strength, the shape and structure of concrete tend to deteriorate and collapse completely. In the case of an ICF wall, the structure does not collapse even after it reaches its peak load. While loading, the ICF wall tends to compress and regain its original shape after the load release; this might be attributed to the presence of the EPS periphery (which comprises 98% air and 2% polystyrene). In conclusion, “the encapsulation of concrete in the EPS system changes the failure type from brittle to ductile.”

**Figure 11:**
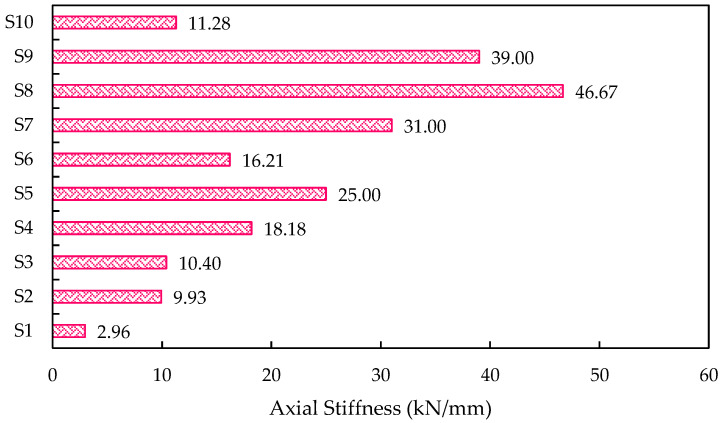
The axial stiffness of the ICF walls.

**Figure 12:**
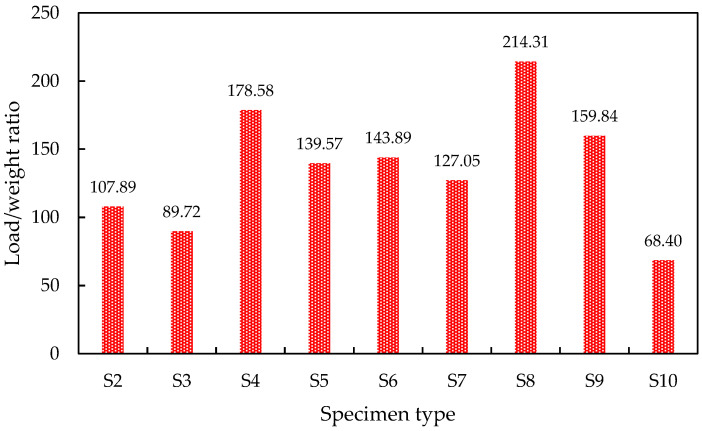
The load-to-weight ratio of the ICF wall panels.

In the case of both M15 and M20 mixes, cracks developed at the top of the specimen, and the specimens failed due to crushing. Under uniform loading, all the ICF panels exhibited significant resistance to compressive stress. The projected cells of panels have shown their failure under compressive loading. In both cases (M15 and M20 mix), the initial crack started at a load of 25 kN, and further, the cracks tend to develop at the consequent loads till failure. Figure 11, shows the axial stiffness of the ICF wall. In addition to the load-deflection parameter, load to weight ratio, was estimated and is shown in Figure 12. The weight of the individual ICF wall panel was found in terms of kg and concerted into Newtons for better assessment. It is found that S4 and S8 possess the maximum value; this shows that S4 and S8 have higher efficiency compared with all other panel types.

### 3.4. Strain Energy

**Figure 13:**
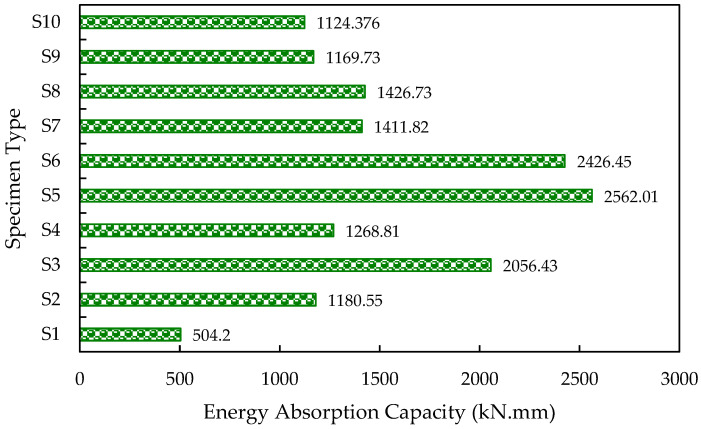
Energy-absorbing capacity of the ICF-based wall panels.

The strain energy is the energy absorbed by a body while undergoing deformation. The strain energy is an indirect indicator used to evaluate ductility. The area under the load-displacement characteristic curve is used to evaluate the ductility. The absorption of energy is related to the ductility of the material. Because the ICF wall panel absorbs more energy than the reference panel, it implicitly reflects ICF’s superior ductility. Equation (1) may be used to determine the energy absorption (EA) of a wall panel [12].

(1) $$\mathrm{Energy}\;\mathrm{Absorption}\;\mathrm{capacity}\;(\mathrm{EAC})=V\int_{0}^{\varepsilon_{c}}\sigma d\varepsilon$$

where V is the specimen volume in cubic meters, ε_c_ is compressive strain, and σ is compressive stress. The compressive load-displacement curve may also be used to calculate energy absorption. The area represents the absorbed strain energy by the wall panel under the compressive load-displacement curve [12]. The ICF wall panels’ high strain energy indicates their high ductility compared with the reference panels. Figure 13 illustrates the energy-absorbing capacity of the wall panels.

### 3.5. Crack Pattern

**Figure 14:**
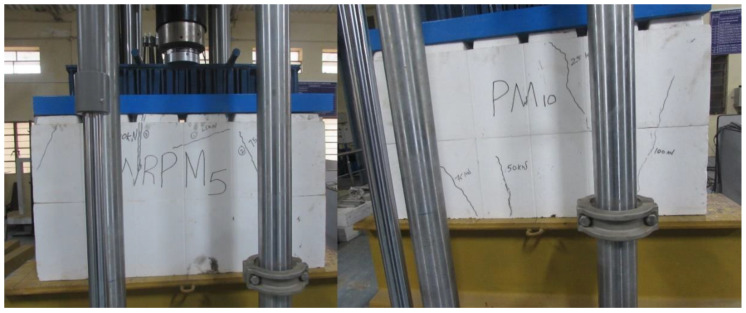
Failure caused while loading.

**Figure 15:**
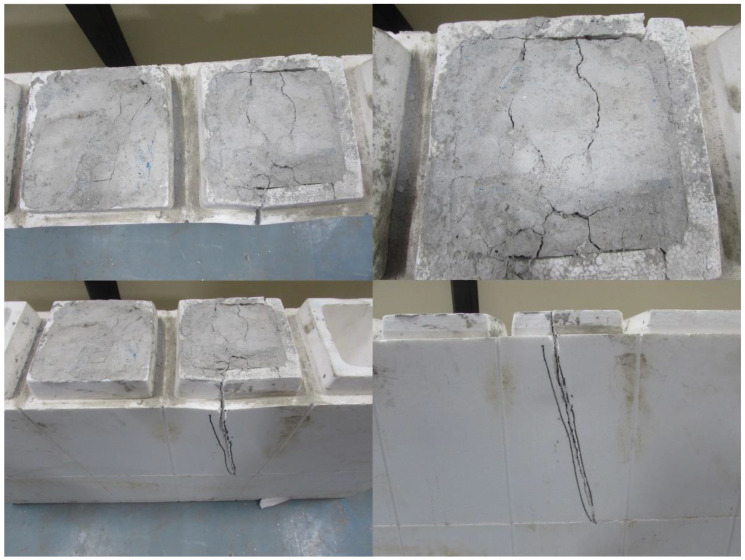
Failure pattern of ICF panels.

Due to the elastic nature of the EPS shell, cracks were not observed in all the ICF-based wall panels. Many minor cracks were seen on the bottom side of the ICF wall panels, then the top surface of the wall panels, as seen in Figure 14 and Figure 15. One or two major cracks were observed in the top surface of the ICF wall panels. Almost all the ICF-based wall panels possess similar failure and crack patterns; the presence of an EPS shell could be attributed to this. Notably, the degradation in load capacity is mainly due to the crushing of concrete in the cells, but the shielding of EPS can enhance the load-carrying capacity further. The final reduction in the load-carrying capacity of panels is due to the tearing of EPS at the bottom phase.

It is observed that the ICF wall panels exhibit the ability to resist the load even after the failure of the concrete core. However, a sudden failure was observed in the reference panel. The reference panel broke into pieces after failure, which is presented in the ICF wall panels because of the elastic nature of the EPS sheets enveloping them. This elastic property of EPS holds the concrete core in position even after its failure. Brittle failure was observed in the reference panel. ICF does not exhibit any such failure; instead, it displays an elastic nature when the axial compressive load is applied. Moreover, after removing the load, EPS in ICF holds together the failed concrete and prevents the collapse of failed concrete when it reaches its peak load.

### 3.6. Cost Efficiency

**Figure 16:**
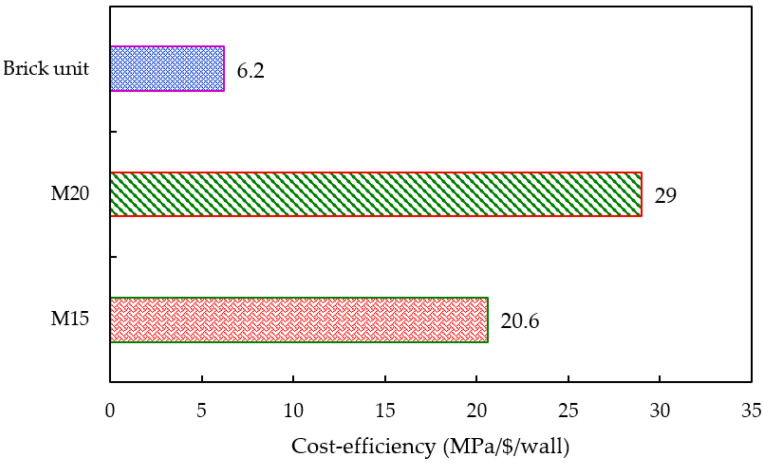
Cost-efficiency analysis of ICF and brick walls.

ICF and brick walls’ cost efficiency is measured by taking the strength-to-cost ratio [39]. The cost involved in this analysis is considered based on the materials’ purchase cost. All the raw ingredients are obtained from local dealers in Coimbatore. The cost of the raw materials is found to be: 130 USD/MT for cement; 67.88 USD/MT for GGBS; 7.31 USD/MT for M-sand; 9.14 USD/MT for coarse aggregate; and 13 USD/MT for FA. The production cost of ICF panels and brick walls is marginal. The early age of GPC is relatively higher than conventional cement-based concrete. The strength-to-cost ratio of the ICF and brick wall is illustrated in Table 5. The higher the value of cost efficiency, the higher the effectiveness in its production. M20 grade concrete mix possesses higher strength than M15 grade mix. This can be attributed to the higher percentages of GGBS content in it. The production cost of the ICF panels and their counterpart brick walls is marginal; this can be attributed to alkaline activators, sodium hydroxide and sodium silicate solutions. Figure 16 illustrates the cost-efficiency analysis of M15 and M20 grade GPC specimens.

The cost-efficiency analysis shows that the ICF-based system provides higher effectiveness in terms of its cost of production. It is concluded that ICF wall panels can be used as alternatives to conventional brick-based walls to enhance the construction speed and lower the production cost.

### 3.7. Advantages of ICF over Masonry Walls

ICF does not require skilled construction labor, so the labor cost is considerably lower. The ICF construction time of the ICF wall is reduced by 50%, and an increase in the construction unit decreases the labor cost. Due to the interconnectivity of EPS with the aid of reinforcement, ICF has higher resistance against static loads. ICF construction in hot and cold regions can reduce power consumption costs due to high heat insulation. Plumbing and electrical ducts can be installed more easily in ICF than in conventional masonry units. ICF can reduce noise as a sound barrier between rooms and create a better living environment. ICF walls are more cost efficient than traditional masonry units. EPS is a recycled material; therefore, the construction of structures using ICF can be considered under green construction practices.

## 4. Conclusions

The performance of encapsulated concrete EPS wall panels under axial compression was studied in the current investigation. The major conclusions of the findings are reported below:

- The encapsulation of concrete in the EPS system changes the failure type from brittle to ductile, and the elastic property of the EPS holds the encapsulated concrete in the core even after the failure;
- The result from the study reveals that Specimen S9 (all the cells of EPS were filled with concrete, M20) exhibits a load of 360 kN with deformation of 11 mm more than the reference masonry unit and all other models;
- In comparison with the reference wall panel, Specimen S2, the load-carrying capacity of Specimen S2 was 3.33% less, whereas the Specimens S3, S4, S5, S6, S7, S8, and S9 gained an increase of 60%, 87%, 57%, 107%, 133%, and 160%, respectively;
- Due to the EPS outer shell, the wall panels possess higher deformations than the conventional brick masonry wall;
- The brick masonry walls exhibit brittle failure; without any sign, the masonry wall fails into pieces, whereas the ICF wall panels retain their shape even after failure. Therefore, ICF can be recommended to replace the conventional load-bearing and non-load-bearing walls;
- Research on the carbon and energy-effective mix design proportion of GPC-based ICF to attain target strength, workability, durability, and sustainability may be carried out. Further investigations are required to elaborately explain the failure pattern, loading-deflection behavior, material characterization of EPS, and the infilling materials so that national standards/guidelines can be developed.

## Figures and Tables

**Table 1:** The oxide composition of FA and GGBS.

Oxides	FA (%)	GGBFS (%)
SiO_2_	61.2	42.4
Al_2_O_3_	26.9	13.2
Fe_2_O_3_	6.21	1.12
CaO	1.91	41.2
MgO	0.29	1.3
Na_2_O	0.58	0.3
K_2_O	1.21	0.7
Ti	0.4	-

**Table 2:** Properties of the ICF wall.

Specification	Material Properties
Size of the ICF wall	20.32 cm (8 inches) × 30.50 cm (12 inches) × 121.00 cm (48 inches)
Water vapor Permeability	0.0020 ng·s·m^2^·Pa
Moisture Absorption (humidity):% mass	0.4417
Moisture Absorption (humidity):% volume	0.0099

**Table 3:** Mix ratios employed in the current investigation.

Type of Mix	Binder		Aggregate (kg/m^3^)		Alkaline Activator (kg/m^3^)		Molarity (M)
	(kg/m^3^)						
	FA	GGBS	M-Sand	Coarse	NH	NS	
M15	180	20	450	650	28.96	43.44	4
M20	100	100	450	650	28.96	43.44	4

**Table 4:** Details of the ICF specimens.

Specimen Type	Reinforcement Type	Specimen Description	Type of Mix
S1	(Without reinforcement)	Hollow EPS without concrete	-
S2		Alternative cells of EPS were filled with concrete	M15
S3		All the cells of EPS were filled with concrete	M15
S4		Alternative cells of EPS were filled with concrete	M20
S5		All the cells of EPS were filled with concrete	M20
S6	(With reinforcement)	Alternative cells of EPS were filled with concrete	M15
S7		All the cells of EPS were filled with concrete	M15
S8		Alternative cells of EPS were filled with concrete	M20
S9		All the cells of EPS were filled with concrete	M20
S10		Masonry (reference)	-

**Table 5:** Cost-efficiency analysis of ICF and brick walls.

S.No	Material		ICF				Brick Wall	
			M15		M20			
		Rate	QTY	Cost	QTY	Cost	QTY	Cost
		(USD/MT)	(1 Wall)	(USD/Wall)	(1 Wall)	(USD/Wall)	(1 Wall)	(USD/Wall)
1	Cement	134.91	-	-	-	-	30	4.05
2	FA	13.49	180	2.43	100	1.35	-	-
3	GGBS	78.6	20	1.57	100	7.86	-	-
4	CA	9.44	650	6.14	350	3.30	-	-
5	MS	7.55	450	3.40	450	3.40	60	0.45
6	SS	121.41	43.44	5.27	43.44	5.27	-	-
7	SH	303.33	28.96	8.78	28.96	8.78	-	-
8	Bricks	0.15/brick	-	-	-	-	275	41.25
9	ICF panel	10/panel	2	20	2	20	-	-
10	Total Cost USD/wall			47.59		49.97		45.75
11	28 days compressive strength (MPa)			20.60		29.00		6.20
12	Cost efficiency (MPa/USD/wall)			0.43		0.58		0.14

Note: SH: Sodium hydroxide; SS: Sodium silicate; QTY: Quantity.

## Data Availability

The data presented in this study are available on request from the corresponding author.
